# Spatial modeling of *Borrelia* genospecies in human-biting ticks from the French citizen science programme CiTIQUE

**DOI:** 10.1038/s41598-026-42619-4

**Published:** 2026-04-21

**Authors:** Thierno Madiou Bah, Jonas Durand, Arnaud Cougoul, William Wint, Francesca Dagostin, Isabelle Lebert, Magalie René-Martellet, Thomas Opitz, Xavier Bailly, Pascale Frey-Klett, Karine Chalvet-Monfray

**Affiliations:** 1 University of Clermont Auvergne, INRAE, VetAgro Sup, UMR EPIA, Saint-Genès Champanelle, France; 2 University of Lyon, INRAE, VetAgro Sup, UMR EPIA, Marcy l’Etoile, France; 3 Laboratoire Tous Chercheurs, Université de Lorraine, INRAE, IAM, Nancy, F-54000 France; 4 Environmental Research Group Oxford Ltd, Oxford, UK; 5 Research and Innovation Centre, Fondazione Edmund Mach, San Michele all’Adige, TN Italy; 6 Biostatistics and Spatial Processes (UR 546), INRAE, Avignon, France

**Keywords:** Diseases, Ecology, Ecology, Microbiology, Zoology

## Abstract

In Europe, Lyme borreliosis is the most common vector-borne human disease, mainly caused by *Borrelia afzelii* and *Borrelia garinii*, two species of the *Borrelia burgdorferi* sensu lato (Bbsl) complex transmitted by *Ixodes ricinus*. Assessing the spatial risk of human exposure to these pathogens is essential for efficient public health surveillance, yet conventional monitoring often fails to produce geographically explicit, large-scale data capturing exposure heterogeneity and its drivers. Focusing on continental France, we used data from the CiTIQUE citizen science programme to analyse spatial variation of Bbsl infection in georeferenced human-biting ticks and to model its associations with environmental, ecological, and anthropogenic factors. From 2017 to 2019, 1,891 ticks were analysed, with 15% testing positive for Bbsl. The most prevalent genospecies were *B. afzelii* (7.2%) and *B. garinii* (4.2%), showing distinct spatial patterns. *I. ricinus* habitat suitability was the most consistent predictor of Bbsl infection probability. Genospecies-specific models highlighted host influences: *B. afzelii* occurrence increased with rodent species richness, whereas *B. garinii* was associated with Turdidae species and showed possible dilution effect by rodents. Our findings demonstrate the value of citizen science in complementing surveillance and provide large-scale, spatially explicit insights into Bbsl eco-epidemiology in France, offering an adaptable framework for vector-borne disease monitoring.

**Supplementary Information:**

The online version contains supplementary material available at 10.1038/s41598-026-42619-4.

## Introduction

Lyme borreliosis (Lb) is the most prevalent vector-borne human disease in Europe, with approximately 129,000 cases reported annually from national surveillance systems^1^. However, as Lb is not a notifiable disease in most European countries, these reports probably underestimate the true incidence. In France, the routine surveillance system estimated approximately 39,000 cases in 2023, but recent analyses of data from a computerised physician decision support system suggest that the actual incidence may have been closer to 191,000 cases during the same year^2^.

Lyme borreliosis is a zoonotic disease caused by pathogenic spirochetes of the *Borrelia burgdorferi* sensu lato (Bbsl) species complex, primarily transmitted by the hard tick *Ixodes ricinus*^3,4^. In Europe, *Borrelia afzelii* and *Borrelia garinii* are the most common genospecies^5^, predominantly maintained by rodents and birds, respectively^6^. Their distinct eco-epidemiological cycles, combined with environmental and anthropogenic factors drive spatial heterogeneity in Bbsl distribution^7^. Since genospecies differ in clinical outcomes, *B. garinii* being more often linked to neuroborreliosis^8^, mapping this variation is critical. Human risk likewise varies widely across landscapes, underscoring the need to map this heterogeneity for surveillance, prevention, and public health actions.

Assessing spatial Lb risk typically relies on two layers of information : acarological hazard, defined by the density of host-seeking Bbsl-infected *I. ricinus* nymphs, and human exposure, often approximated by clinical findings^5,9^. Both are essential for modelling the local states of the host–vector–pathogen system and predicting infection risk^10^. Two complementary approaches are used: mechanistic models, which explicitly represent life-cycle and transmission processes^11^, and statistical models^12^, which link infection patterns to environmental or socio-economic factors^10,13^.

Statistical models can target specific components, such as the spatial distribution of Bbsl in ticks, rather than full system dynamics. This focus allows the use of large-scale, spatially explicit data, whereas mechanistic models require extensive parameterization for which data is often difficult and costly to obtain due to the system complexity.

Field methods, such as ticks sampling from hosts, provide insights into pathogen ecology but rarely capture the full diversity of reservoirs within a single study (e.g.^14^., for birds in France). Standardised questing tick sampling offers a good proxy for tick density and pathogen hazard (see for instance^15,16^. However, tick sampling is often not standardized at country level, is limited to small areas or accessible environments and lacks information on human exposure. Human case reports from medical practitioners can provide broader spatial coverage of human risk of infection but are often limited by coarse spatial resolution^17^.

To overcome these limitations, citizen science initiatives have emerged as promising tools to generate geographically informed, large-scale data on human-tick encounters. Such data would otherwise be extremely difficult, if not impossible, to obtain through conventional research means, while also fostering engagement and raising awareness^18^. In particular, the collection of human-biting ticks is directly associated to human exposure^19^, and can be used to study spatial variations in the probability of Bbsl genospecies infection in human-biting ticks.

France hosts diverse climates and environments^20^ that support both tick populations and pathogen reservoirs^15,16,21^. Previous studies on Bbsl distribution drivers in France were conducted at highly localised scales^22,23^, making extrapolation to the contiguous national level difficult. At the other end of the spectrum, European-scale models of Bbsl distribution have been developed^7^, but these rely on literature data which are from France limited and at a very local scale, highlighting the need for extensive data and studies to better understand and predict Bbsl risk in this country suffering high Lyme borreliosis incidence^24^.

Here, we used human-biting ticks collected through the CiTIQUE citizen-science programme between 2017 and 2019, to study the spatial distribution of Bbsl and its major genospecies across continental France. Combining statistical relative risk mapping, which quantifies the relative density of pathogen presence in relation to its absence, and generalised additive models (GAMs), we characterised the spatial heterogeneity of tick infection risks and identified environmental and ecological drivers shaping the distribution of Bbsl in human-biting ticks, providing a detailed, data-driven picture of Lb eco-epidemiology across continental France.

## Results

### Descriptive analysis

A total of 1,891 human-biting *Ixodes ricinus* ticks were screened for pathogens, of which 291 (15%) were found to be infected with *Borrelia burgdorferi* sensu lato (Bbsl). Among these, *Borrelia afzelii* and *Borrelia garinii* were the most prevalent genospecies, infecting 136 (7.2%) and 80 (4.2%) individual ticks, respectively (Table 1). Infections with other genospecies were less common, with 37 ticks (2%) infected with *Borrelia valaisiana*, 25 (1.3%) with *Borrelia burgdorferi* sensu stricto, 8 (0.4%) with *Borrelia spielmanii*, and 5 (0.3%) with *Borrelia lusitaniae*. No ticks were found to be infected with *Borrelia bissettii* (Table 1).

**Table 1:** Count and prevalence of *Borrelia burgdorferi* sensu lato genospecies found in human-biting ticks per region. Prevalence are in percentage and 95% confidence interval are between brackets. n: total number of analyzed ticks in each region. Region: ARA: Auvergne-Rhône-Alpes; BFC: Bourgogne-Franche-Comté; BRE: Bretagne; CVL: Centre-Val de Loire; GES: Grand-Est; IDF: Île-de-France; NOR: Normandie; NAQ: Nouvelle-Aquitaine; OCC: Occitanie; PDL: Pays-de-la-Loire.

Borrelia genospecies	Regions											
	ARA *n* = 166	BFC *n* = 157	BRE *n* = 224	CVL *n* = 149	GES *n* = 298	HDF *n* = 159	IDF *n* = 156	NOR *n* = 150	NAQ *n* = 174	OCC *n* = 148	PDL *n* = 166	PAC *n* = 59
Borrelia burgdorferi sensu lato	28; 16.9% (11.5–23.4)	30; 19.1% (13.3–26.1)	26; 11.6% (7.7–16.5)	29; 19.5% (13.4–26.7)	60; 20.1% (15.7–25.1)	20; 12.6% (7.9–18.8)	21; 13.5% (8.5–19.8)	12; 8% (4.2–13.6)	29; 16.7% (11.5–23.1)	18; 12.2% (7.4–18.5)	17; 10.2% (6.1–15.9)	1; 1.7% (0-9.1)
Borrelia afzelii	16; 9.6% (5.6–15.2)	19; 12.1% (7.4–18.3)	5; 2.2% (0.7–5.1)	11; 7.4% (3.7–12.8)	31; 10.4% (7.2–14.4)	12; 7.5% (4-12.8)	5; 3.2% (1-7.3)	3; 2% (0.4–5.7)	12; 6.9% (3.6–11.7)	13; 8.8% (4.8–14.6)	8; 4.8% (2.1–9.3)	1; 1.7% (0-9.1)
Borrelia garinii	7; 4.2% (1.7–8.5)	3; 1.9% (0.4–5.5)	15; 6.7% (3.8–10.8)	11; 7.4% (3.7–12.8)	16; 5.4% (3.1–8.6)	3; 1.9% (0.4–5.4)	6; 3.8% (1.4–8.2)	7; 4.7% (1.9–9.4)	8; 4.6% (2-8.9)	1; 0.7% (0-3.7)	3; 1.8% (0.4–5.2)	0; 0% (0-6.1)
Borrelia valaisiana	3; 1.8% (0.4–5.2)	3; 1.9% (0.4–5.5)	3; 1.3% (0.3–3.9)	5; 3.4% (1.1–7.7)	7; 2.3% (0.9–4.8)	2; 1.3% (0.2–4.5)	5; 3.2% (1-7.3)	1; 0.7% (0-3.7)	3; 1.7% (0.4-5)	4; 2.7% (0.7–6.8)	1; 0.6% (0-3.3)	0; 0% (0-6.1)
Borrelia burgdorferi sensu stricto	1; 0.6% (0-3.3)	3; 1.9% (0.4–5.5)	3; 1.3% (0.3–3.9)	1; 0.7% (0-3.7)	4; 1.3% (0.4–3.4)	2; 1.3% (0.2–4.5)	2; 1.3% (0.2–4.6)	1; 0.7% (0-3.7)	4; 2.3% (0.6–5.8)	0; 0% (0-2.5)	4; 2.4% (0.7–6.1)	0; 0% (0-6.1)
Borrelia lusitaniae	1; 0.6% (0-3.3)	0; 0% (0-2.3)	0; 0% (0-1.6)	1; 0.7% (0-3.7)	0; 0% (0-1.2)	0; 0% (0-2.3)	1; 0.6% (0-3.5)	0; 0% (0-2.4)	1; 0.6% (0-3.2)	0; 0% (0-2.5)	1; 0.6% (0-3.3)	0; 0% (0-6.1)
Borrelia spielmanii	0; 0% (0-2.2)	2; 1.3% (0.2–4.5)	0; 0% (0-1.6)	0; 0% (0-2.4)	2; 0.7% (0.1–2.4)	1; 0.6% (0-3.5)	2; 1.3% (0.2–4.6)	0; 0% (0-2.4)	1; 0.6% (0-3.2)	0; 0% (0-2.5)	0; 0% (0-2.2)	0; 0% (0-6.1)

**Fig. 1:**
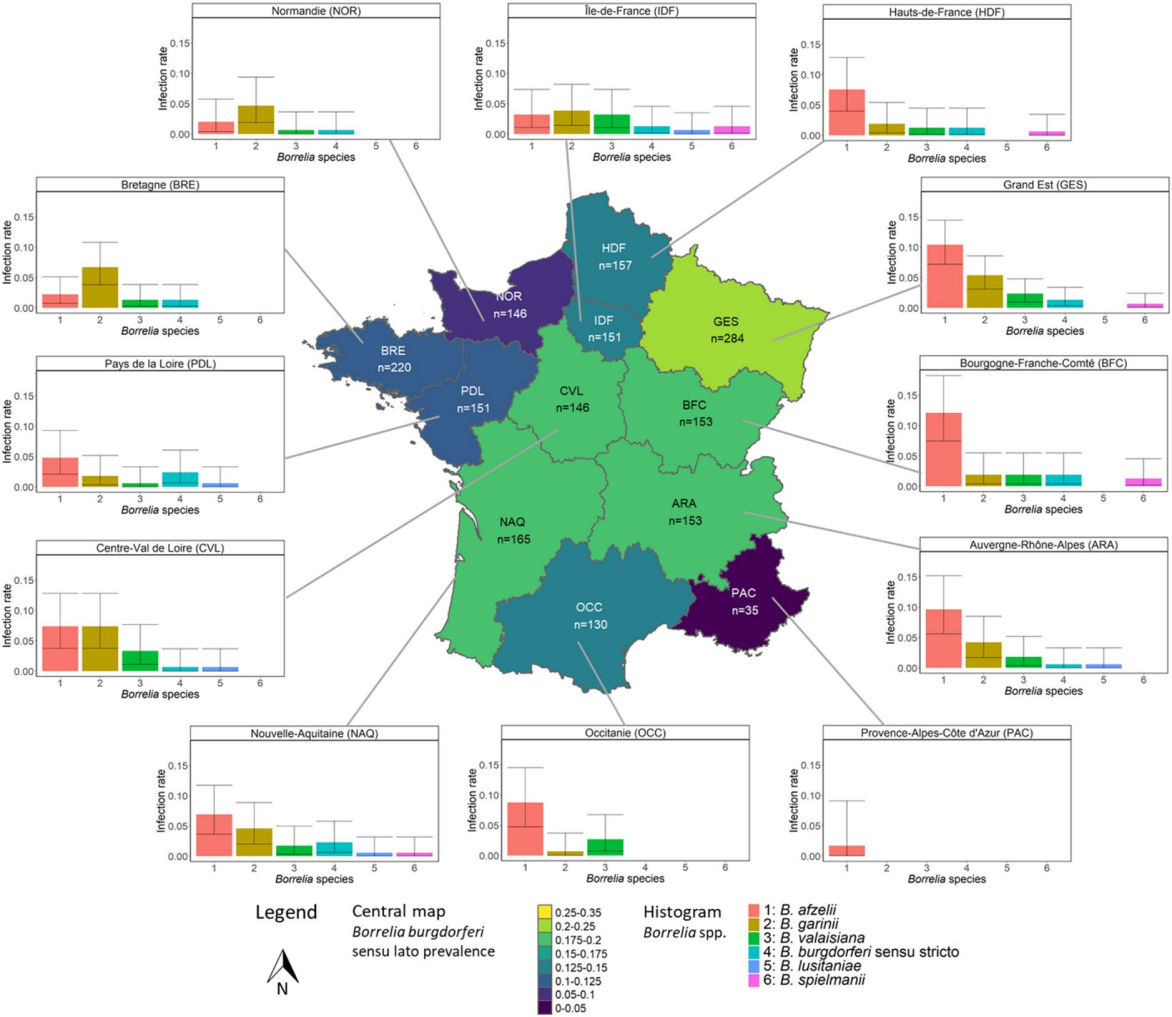
Prevalence of *Borrelia burgdorferi* sensu lato per region in analyzed human-biting *Ixodes ricinus* ticks (central map), and histograms of the specific prevalence of the main Borrelia species in each region. Central map: n: number of *I. ricinus* ticks analyzed in the considered region. Histogram: Confidence interval 95%. ARA: Auvergne-Rhône-Alpes; BFC: Bourgogne-Franche-Comté; BRE: Bretagne; CVL: Centre-Val de Loire; GES: Grand-Est; HDF: Hauts-de-France; IDF: Île-de-France; NAQ: Nouvelle-Aquitaine; NOR: Normandie; OCC: Occitanie; PAC: Provence-Alpes-Côte d’Azur; PDL: Pays-de-la-Loire. Reproduced from Durand et al.^25^ under a Creative Commons Attribution (CC-BY) license.

*I. ricinus* ticks were collected across the continental French territory, and Bbsl was detected in all NUTS-1 regions. However, the spatial distribution of infected ticks, was uneven (Fig. 1; Table 1, Supplemental Fig. 1) Durand et al.^25^ identified four regional group, of which the high prevalence group including Auvergne-Rhône-Alpes (ARA), Bourgogne-Franche-Comté (BFC), Centre-Val de Loire (CVL), and Grand Est (GES) had a higher number of infected sample relative to sampling effort compared the other regional groups (OR = 1.62, *p* = 0.004; OR = 2.27, *p* < 0.001; and OR = 13.98, *p* = 0.045, respectively).

### Factors associated with Bbsl distribution

**Fig. 2:**
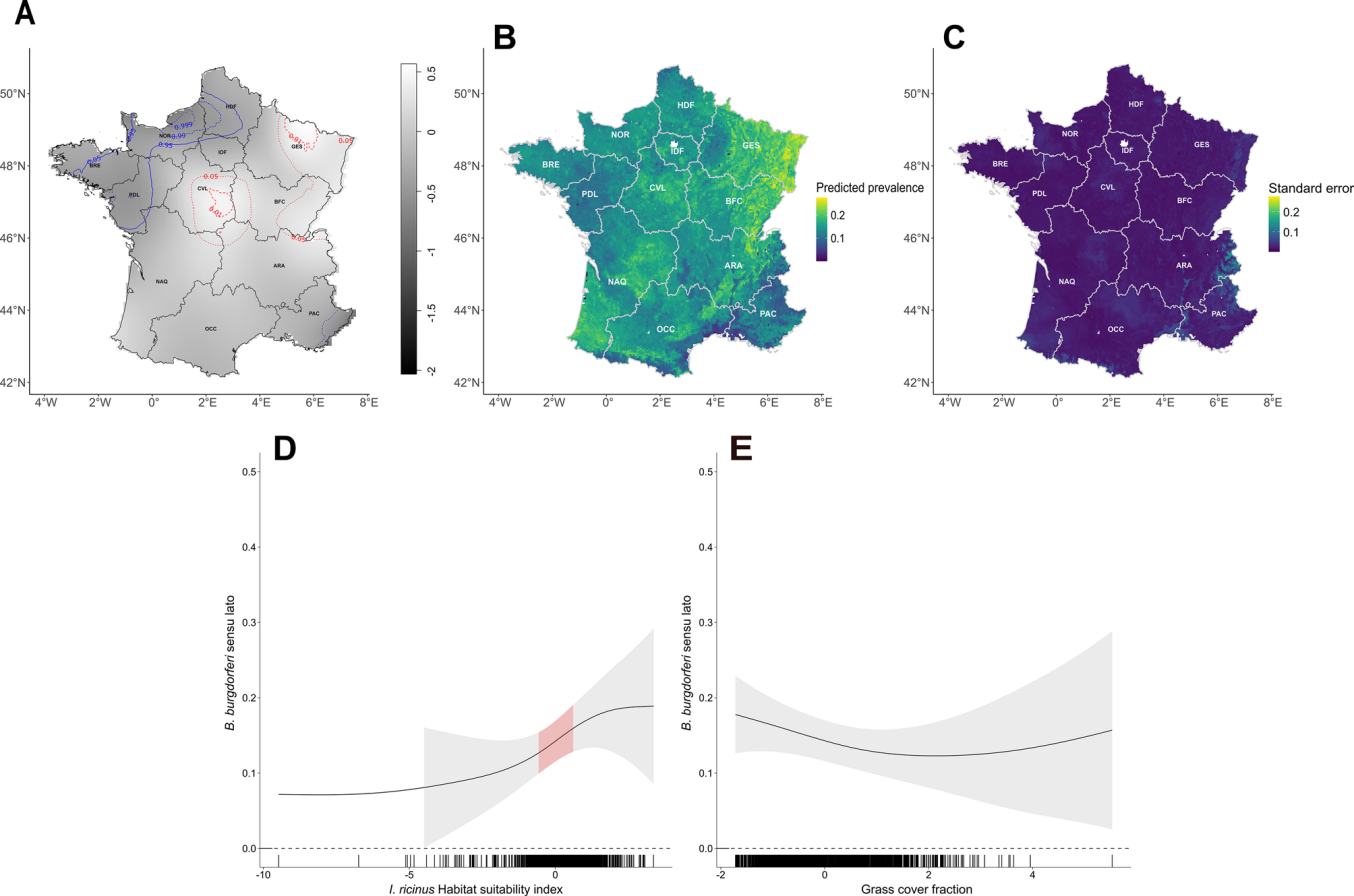
Model predictions for *Borrelia burgdorferi sensu lato* (Bbsl) prevalence in *Ixodes ricinus* tick across France. (**A**) Bbsl relative risk surface using the Bbsl presence and absence data. Tolerance contours represent significantly lower risk areas in blue and higher risk areas in red. (**B**) Predicted prevalence of Bbsl based on the M0 GAM, expressed as the predicted proportion of Bbsl infected ticks (range 0–1), with values ranging from low (dark blue) to high (yellow-green) prevalence. (**C**) Corresponding standard error of the GAM predictions, with lower uncertainty indicated in dark blue and higher uncertainty in yellow. (**D**,**E**) Each plot demonstrates the marginal effects of *I. ricinus* habitat suitability index (**D**) and grass cover fraction (**E**) on Bbsl predicted prevalence, with 95% confidence intervals shown in shaded areas while values for which the slope is significantly different from zero are highlighted in red. Black ticks along the x-axis represent observed values of the covariables.

The results of the first GAM model (M0), which investigated factors associated with Bbsl prevalence, are presented in Fig. 2. After penalisation, only two variables were retained as significant predictors: the *I. ricinus* habitat suitability index and the grass cover fraction (Table 2). The *I. ricinus* suitability index showed a positive association with Bbsl prevalence (edf = 1.560; χ² = 6.558; p-value = 0.007), indicating that higher *I. ricinus* habitat suitability values correspond to an increased probability of Bbsl infection, with this relationship plateauing at higher suitability values (Fig. 2D). Bbsl prevalence exhibited a convex relationship with grass cover fraction (edf = 1.104; χ² = 3.445; p-value = 0.026). At lower grass cover values, an initial increase was associated with a decline in Bbsl prevalence, reaching a minimum, followed by a slight increase at higher grass cover values. However, this latter trend was characterised by greater uncertainty, likely due to the limited number of observations in areas with high grass cover (Fig. 2E).

The relative risk map (Fig. 2A) highlights significant low-risk areas (blue contours), which include the Bretagne (BRE) and Normandie (NOR) regions in northwestern France, and significant high-risk areas (red contours), concentrated in the Grand Est (GES), Bourgogne-Franche-Comté (BFC), and Centre-Val de Loire (CVL) regions in the east and centre of the country. Model predictive performance was low (mean AUC = 0.56 based on 100 repetitions of 90/10 cross-validation). Nevertheless, high-risk regions identified by the relative risk map correspond to areas with higher Bbsl prevalence predicted by the GAM model (Fig. 2B). Conversely, BRE and NOR exhibited lower predicted prevalence, consistently with the low-risk areas identified by the relative risk analysis. Additional regions, particularly in the southwest and Auvergne-Rhône-Alpes (ARA), also displayed elevated Bbsl prevalence according to the GAM model predictions (Fig. 2B). The associated uncertainty in prevalence predictions was highest in the Rhône Valley (southeastern France) and the Alpine regions (eastern France) where sample density was lower and environmental heterogeneity may be greater (Fig. 2C).

### Factors associated with *Borrelia afzelii* distribution

**Fig. 3:**
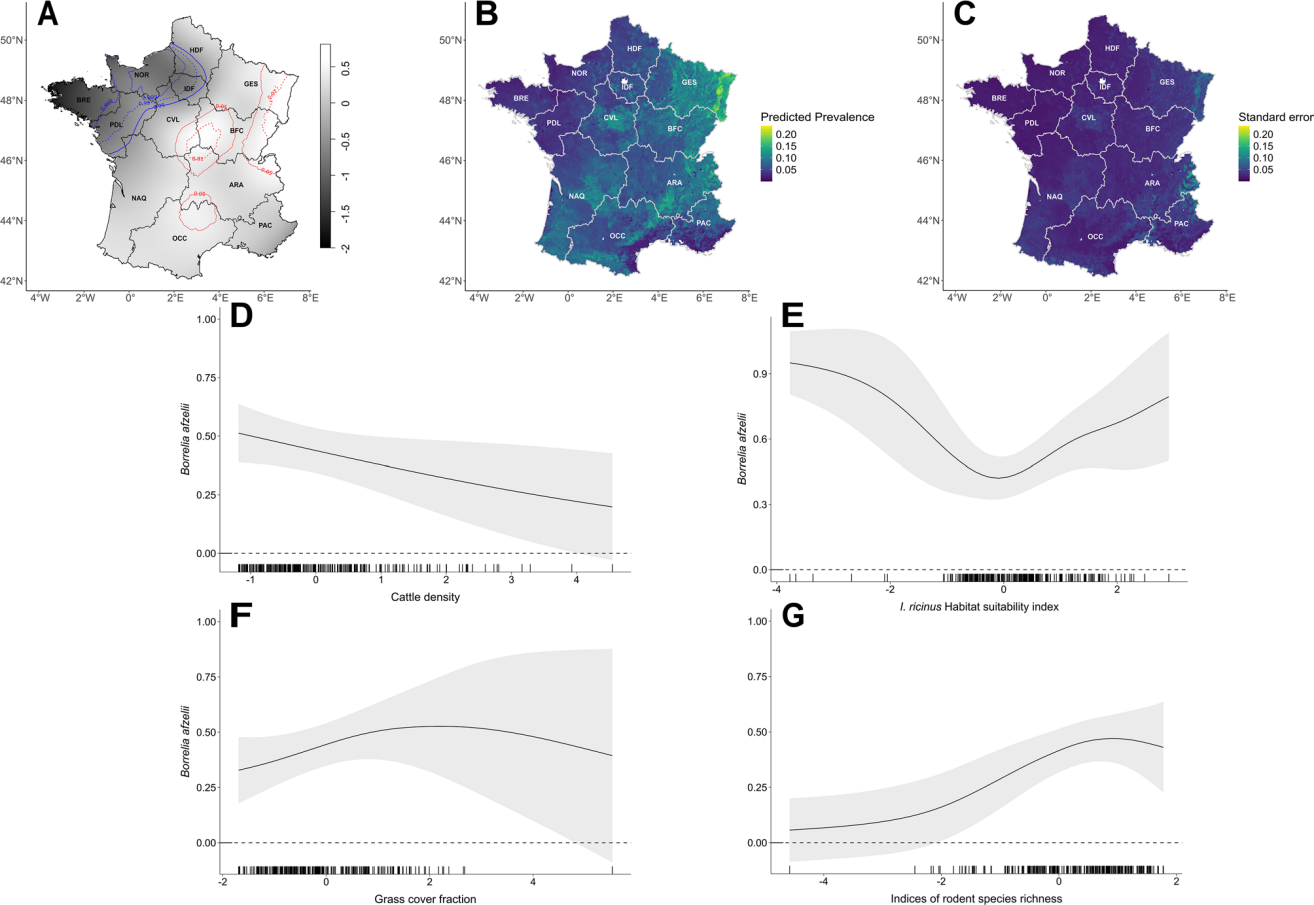
Model predictions for *Borrelia afzelii* prevalence in *Ixodes ricinus* tick across France. (**A**) *B. afzelii* relative risk surface using the *B. afzelii* presence and absence data. Tolerance contours represent significantly lower risk areas in blue and higher risk areas in red. (**B**) Predicted prevalence of *B. afzelii* based on the product of the predicted general *Borrelia burgdorferi sensu lato* (Bbsl) presence (M0) with the *B. afzelii* relative probability knowing presence (M1). Prevalence values are expressed as the predicted proportion of *B. afzelii* infected ticks (range 0–1), ranging from low (dark blue) to high (yellow-green) prevalence. (**C**) Corresponding standard error of the predicted prevalence of *B. afzelii* based on the product of M0 and M1, with lower uncertainty indicated in dark blue and higher uncertainty in yellow. (**D**–**G**) Each plot demonstrates the marginal effects of the model on the relative probability of having *B. afzelii* knowing Bbsl presence (M1), with Cattle density (**D**), *I. ricinus* habitat suitability index (**E**), indices of rodent species richness (**F**) and grass cover fraction (**G**). 95% confidence intervals are shown in shaded areas while values for which the slope is significantly different from zero are highlighted in red. Black ticks along the x-axis represent observed values of the covariables.

The results of the M1 GAM model assessing factors associated with *B. afzelii* distribution are presented in Fig. 3. This model focused on sites where a *Borrelia* genospecies was detected, with the presence probability of *B. afzelii* (conditional on overall *Borrelia* presence) as the response variable. Therefore, the model highlights determinants of the variability in relative incidence of *B. afzelii* among all *Borrelia* occurrences. Four covariates were identified as significant predictors: cattle density, *I. ricinus* habitat suitability index, rodent species richness, and grass cover fraction.

Cattle density was negatively associated with *B. afzelii* presence probability (edf = 0.753; χ² = 2.808; p-value = 0.035), although uncertainty increased at higher cattle densities (Fig. 3D). The relationship between *B. afzelii* presence and *I. ricinus* habitat suitability index was convex (edf = 2.697; χ² = 9.142; p-value = 0.009), with a decline in presence probability observed at lower suitability values, reaching a minimum around 0 on the x-axis, followed by an increase at higher suitability values. However, both trends were accompanied by high uncertainty (Fig. 3E). A concave relationship was observed with rodent species richness (edf = 1.788; χ² = 6.618; p-value = 0.015), peaking around a richness value of one, before slightly declining. Uncertainty was highest at both low and high richness values (Fig. 3F). Grass cover fraction also showed a concave relationship (edf = 1.041; χ² = 2.959; p-value = 0.041), with *B. afzelii* presence probability increasing to a maximum at intermediate grass cover values (around 2 on the x-axis), followed by a slight decline and higher uncertainty at greater grass cover fractions (Fig. 3G).

The relative risk map (Fig. 3A) revealed that areas of significant low (blue contours) and high (red contours) *B. afzelii* risk broadly overlapped with those identified for Bbsl. However, an additional high-risk area was detected in the northern part of the Occitanie (OCC) region. Model predictive performance was moderate (mean AUC = 0.62 based on 100 repetitions of 90/10 cross-validation), yet predictions from the GAM aligned with the relative risk surface, with the highest *B. afzelii* prevalence in northeastern regions, including Grand Est (GES), Bourgogne-Franche-Comté (BFC), and Centre-Val de Loire (CVL). High infection risk was also predicted in regions such as Nouvelle-Aquitaine and Auvergne-Rhône-Alpes (Fig. 3B). Model predictive performance was moderate, with the 100-iteration of 90/10 cross-validation yielding a mean of 0.62. Prediction uncertainty was greatest in the Rhône Valley (southeastern France), the Alps (eastern France), and the eastern part of the GES region, which also correspond to areas of high predicted prevalence (Fig. 3C).

### Factors associated with *Borrelia garinii* distribution

The predicted prevalence of *B. afzelii* and *B. garinii* was calculated by the product of the predicted general Bbsl presence (M0) with the species-specific relative probability (M1 or M2 depending on the genospecies).

**Fig. 4:**
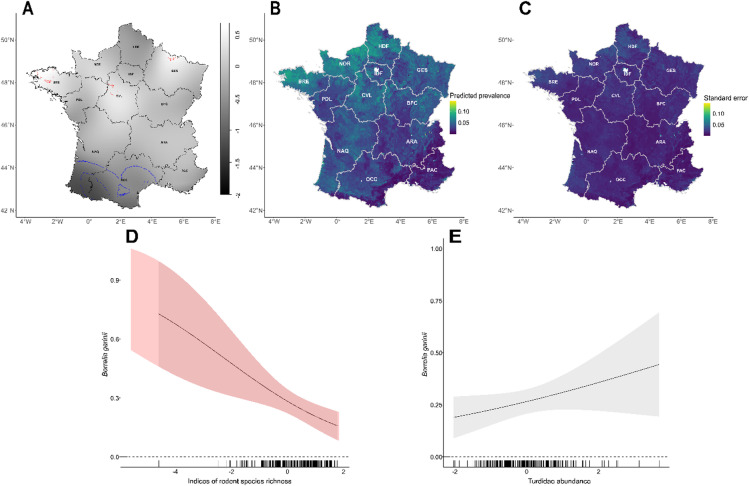
Model predictions for *Borrelia garinii* prevalence in *Ixodes ricinus* tick across France. (**A**) *B. garinii* relative risk surface using the *B. garinii* presence and absence data. Tolerance contours represent significantly lower risk areas in blue and higher risk areas in red. (**B**) Predicted prevalence of *B. garinii* based on the product of the predicted general *Borrelia burgdorferi sensu lato* (Bbsl) presence (M0) with the *B. garinii* relative probability knowing presence (M2). Prevalence values are expressed as the predicted proportion of B. garinii ticks (range 0–1), ranging from low (dark blue) to high (yellow-green). (**C**) Corresponding standard error of the predicted prevalence of *B. garinii* based on the product of M0 and M2, with lower uncertainty indicated in dark blue and higher uncertainty in yellow. (**D**–**F**) Each plot shows the marginal effects of the model on the relative probability of having *B. garinii* knowing Bbsl presence (M2) with indices of rodent species richness (**D**), *Turdidae* abundance (**E**) on the probability of having *B. garinii* knowing Bbsl presence. 95% confidence intervals are shown in shaded areas while values for which the slope is significantly different from zero are highlighted in red. Black ticks along the x-axis represent observed values of the covariables.

The results of the M2 GAM assessing factors associated with *B. garinii* distribution are presented in Fig. 4. This model, again restricted to locations where *Borrelia* was detected, used the presence probability of *B. garinii* (conditional on *Borrelia* presence) as the response variable. Therefore, the model highlights determinants of the variability in relative incidence of *B. garinii* among all *Borrelia* occurrences. Two covariates were identified as significant predictors: rodent species richness and *Turdidae* abundance.

Rodent species richness showed a significant negative association with *B. garinii* presence probability (edf = 0.898; χ² = 8.573; p-value = 0.002), with increased uncertainty at lower values along the x-axis (Fig. 4D). In contrast, Turdidae abundance had a significant positive effect on *B. garinii* presence probability (edf = 0.714; χ² = 2.422; p-value = 0.049), although uncertainty increased markedly at higher abundance values (Fig. 4E).

The relative risk map of *B. garinii (*Fig. 4A) revealed localised high-risk areas (red contours) in the western part of the Bretagne (BRE) region and the western part of the Centre-Val de Loire (CVL) region. Low-risk areas (blue contours) were concentrated in the southwestern regions, including Occitanie and Nouvelle-Aquitaine (NAQ) (Fig. 4A). Model predictive performance was low (mean AUC = 0.58 based on 100 repetitions of 90/10 cross-validation), yet GAM model predictions (Fig. 4B) were consistent with the relative risk map, showing the highest *B. garinii* infection probabilities in the identified high-risk areas. Additional suitable areas for *B. garinii* were predicted in northwestern France, particularly in Bretagne (BRE), Normandie (NOR), and Hauts-de-France (HDF), as well as in the northern parts of NAQ and CVL. Uncertainty associated with the infection probability predictions was generally low but increased in areas with the highest predicted infection probability, notably in the high-risk zones highlighted on the map (Fig. 4C).

## Discussion

Assessing spatial risk of Lyme borreliosis is challenging, as localized surveys lack coverage and broad incidence data miss ecological drivers. By leveraging CiTIQUE citizen-science data, our study provides the first geographically explicit, large-scale view of *Borrelia burgdorferi* sensu lato distribution in France, linking infection risk with key environmental, ecological, and anthropogenic factors.

*B. afzelii* and *B. garinii* were the most prevalent genospecies in the biting ticks which is consistent with the dominant genospecies and their relative frequencies reported across European countries, primarily based on questing tick studies^5,26^. However, the pathogen detection method only identified the dominant genospecies in co-infected ticks, potentially underestimating the prevalence of other species. For example, if *B. afzelii* dominates in co-infected ticks, it may have been disproportionately reported in areas with high tick abundance and higher frequencies of co-infection.

Previous work at the European scale^7^, using a rough resolution ( around 28 km × 28 km per cell) reported high prevalence of *B. afzelii* across France, particularly in eastern Auvergne-Rhône-Alpes. In contrast, our analyses at a finer resolution (5 km x5km per cells) revealed a more heterogeneous pattern, with higher prevalence in eastern and central regions (Grand Est, Bourgogne-Franche-Comté, Centre-Val de Loire) and lower rates in western, northern, and southern regions (Bretagne, Normandie, Occitanie). For *B. garinii*, they found very high prevalence in these western, southern and northern regions, whereas our analyses revealed a more even distribution nationwide, while still confirming higher prevalence in the western and northern part.

Our GAMs highlighted distinct environmental and ecological factors associated with the distribution of Bbsl and its two main genospecies, *B. afzelii* and *B. garinii*. At the overall Bbsl level, infection probability was positively associated with the *I. ricinus* habitat suitability index, a composite indicator derived from a multi-criteria decision analysis integrating climate, altitude, land cover and wild ungulates density, which are factors known to influence tick abundance^21^. This result suggests that, in areas where human exposure occurs, favourable environments for ticks also promote higher Bbsl circulation and prevalence. This finding aligns with theoretical models predicting a positive, yet non-linear, relationship between tick density and pathogen prevalence, modulated by host community composition, including the presence of non-competent host for Bbsl transmission^27,28^.

Observational studies in comparable ecological settings are consistent with these results. In Belgium, tick density was identified as a key driver of Bbsl-infected nymph density, with limited evidence for dilution effects from non-competent hosts^29^. Similarly, in the Netherlands, increased ungulate abundance led to higher tick density and a non-linear, accelerating rise in the density of Bbsl-infected nymphs, without clear evidence for dilution effects attributable to ungulates^30^. Long-term monitoring in southern England further showed that habitats favourable to ticks, such as structurally diverse forests and woodland edges, sustain higher densities of both questing and Bbsl-infected *I. ricinus* nymphs^31^.

At the genospecies level, habitat and host associations differed, reflecting the distinct ecological characteristics of *B. afzelii* and *B. garinii*^6,32,33^. *Borrelia garinii* infection probability was positively associated with Turdidae bird abundance, consistent with its known reliance on avian hosts^34,35^, and negatively associated with rodent species richness, possibly reflecting a dilution effect from these non-specific host^30^. Conversely, *B. afzelii* infection probability was positively associated with rodent richness, consistent with the role of small mammals as primary reservoirs for this genospecies^36^. Rodent richness was also positively associated with the presence of Bbsl, likely reflecting both the dominance of *B. afzelii* in our dataset and the association of several minor Bbsl genospecies, as *B. burgdorferi sensu stricto*, with small mammal hosts^37^. However, these results on *B. afzelii* could be either because *B. afzelii* is very dominant especially in the core areas of *Borrelia* presence or it could be a consequence of a method bias toward *B. afzelii* in co infected ticks, linking its presence with areas of high prevalence.

Grass cover exhibited a non-linear association with Bbsl and *B. afzelii* infection probabilities, with a negative trend across most observed values. Elevated risk was observed at low grass cover levels, characteristic of forest, fragmented woodlands, and associated ecotones, including adjacent private gardens, which are all known to favour both tick and *Borrelia* presence^38^. In contrast, extensive grass cover, typical of open meadows or pastures, was associated with lower infection probabilities, likely due to reduced tick survival in open, hot, and dry environments, and a lower density of competent hosts^39,40^.

Our modelling approach prioritised interpretability using a parsimonious set of covariates, selected to limit collinearity and capture the main ecological drivers, while accounting for the current sampling structure. Although additional covariates, such as vegetation indices^7^, forest structural complexity^41^ or soil properties^42^, have been highlighted in previous studies, our sampling resolution likely limits the reliable detection of such fine-scale environmental effects. This limitation is reflected in the modest predictive performance of the models, as indicated by the low AUC values.

Sampling constraints also restricted our capacity to investigate several relevant aspects of Bbsl infection risk, including variation across tick developmental stages, seasonal and inter-annual dynamics, as well as prevalence across specific environmental contexts. Despite efforts to mitigate sampling bias, by analysing a minimum of 150 ticks per region, a substantial proportion of the analysed samples originated from densely populated areas, leaving gaps in remote and sparsely populated regions in France. A further challenge in estimating Lyme disease risk is the limited understanding of the components leading host seeking infected Bbsl tick to human populations^43^. In this regard, our analysis on human-biting ticks provides valuable insights^44^. However, a more precise assessment of risk would require comparison with prevalence in questing ticks, to disentangle ecological hazard from human exposure. Finally, integrating CiTIQUE tick-bite reports could provide complementary information on the socio-behavioral and ecological factors shaping human exposure thereby improving risk mapping^45^.

Despite these limitations, our study demonstrates the substantial potential of citizen-generated data for monitoring tick-borne pathogen distribution at a national scale. The ticks analysed here represent approximately 40% of all human-biting ticks submitted to CiTIQUE along with a tick-bite report during the study period. Since its inception, the CiTIQUE programme has grown considerably, with over 60,000 human and animal-biting ticks currently stored in the national tick bank. This continuously expanding resource offers unique opportunities to refine surveillance and research on tick-borne diseases in France.

As data and biting ticks continues to be collected, targeted sub-sampling strategies could be implemented to improve spatial representativeness, assess temporal dynamics, compare prevalence across tick developmental stages, and address refined ecological questions regarding Bbsl infection risk. Such approaches will contribute to a more detailed understanding of the eco-epidemiology of Bbsl genospecies and other tick-borne pathogens in human-biting ticks.

In the context of global environmental change and potential shifts in ticks and tick-borne pathogen distributions, the continued development of CiTIQUE provides a scalable, adaptable, citizen-driven tool to support large scale surveillance, environmental risk assessment, and public health preparedness.

## Materials and methods

### Tick acquisition

Ticks were collected through the CiTIQUE citizen science programme (www.citique.fr), launched in July 2017 as a collaboration between INRAE (French National Research Institute for Agriculture, Food and Environment), the Laboratory of Excellence ARBRE, Anses (French Agency for Food, Environmental and Occupational Health & Safety), and the CPIE network (Permanent Centre for Environmental Initiatives). This programme aims to improve the understanding of the ecology of ticks and the tick-borne diseases they transmit, particularly Lyme Borreliosis, in order to support prevention efforts based on monitoring.

Citizens can participate in the CiTIQUE programme choosing among various levels of involvement, ranging from promoting the programme to actively contributing to research through activities in the open lab “Tous Chercheurs”, or by reporting tick bites and collecting and sending biting ticks to the tick bank maintained by the national programme. Participants can report tick bites via a website, a mobile application or by using a paper form, providing the date and GPS location of the bite, along with basic personal information (age, sex, activity at the time of the bite, tick localisation on the body) and the ecological characteristics of the place where the tick bite occurred (e.g., forest, garden, meadow…). When a tick was collected, citizens were instructed to enclose it in a piece of kitchen roll and tape it to a sheet of paper before sending it by courier. Upon reception, ticks were conserved in a freezer before identification and linked to their respective tick-bite reports (https://www.citique.fr/signaler-une-piqure/). Between 2017 and 2020, more than 17,000 human tick-bite reports were submitted, with over 4,500 of these associated with one or more tick specimens.

### Pathogen identifications

A total of 2009 ticks, collected between 2017 and 2019 (inclusive), were randomly selected for DNA extraction, with the objective of including at least 150 human-biting ticks for each French NUTS-1 regions (Nomenclature of Territorial Units for Statistics - major socio-economic regions) except for Provence-Alpes-Côte d’Azur (PAC) with 59 records. From this dataset, 1 891 *Ixodes ricinus* ticks were retained for the modelling analyses presented in this study, including 221 adults, 1324 nymphs and 110 larvae, with 236 undetermined specimens. Details on the overall sample, selection procedure, and dataset structure can be found in Durand et al.^25^.

The tick stages and species were identified morphologically using identification keys by Pérez-Eid^46^ and Estrada-Pena et al.^47^. Species identification was confirmed using specific primers and probes on the microfluidic real-time PCR assay described below. Tick DNA was extracted and screened for pathogens using the method described in Melis et al.^48^. Briefly, tick DNA was extracted using NucleoSpin^®^ Tissue kit (Macherey-Nagel, Germany), following manufacturer’s instructions. Then, all samples underwent a pre-amplification step by PCR with the Preamp Master Mix (Standard BioTools, USA). High-throughput microfluidic real-time PCR was then performed on a BioMark™ real-time PCR system (Standard BioTools, USA), using the 48.48 Dynamic Array™ (Standard BioTools, USA) to detect pathogens and identify ticks. Only results for the different Bbsl genospecies are presented and used in this paper: *Borrelia afzelii*, *Borrelia garinii*, *Borrelia burgdorferi* sensu stricto (s.s.), *Borrelia valaisiana*, *Borrelia spielmanii*, *Borrelia bissettii*, *Borrelia lusitaniae*. This method cannot detect coinfections between different *Borrelia* genospecies. The sequences of the primers and probes are provided in Michelet et al.^49^.

### Covariates acquisition

To explain Bbsl spatial distribution, we extracted covariates related to the density of host-seeking *I. ricinus*, pathogen occurrence and persistence, and human exposure. Climatic variables were included for their effect on tick habitat suitability^50^, along with habitat suitability indices for *I. ricinus* and host-related variables. Non-competent hosts (e.g., roe deer) were also considered for their role as tick amplifiers^51^. Because pathogens were identified in human-biting ticks, we further included variables reflecting human exposure, such as population density and human pressure.

In total, 103 covariates were extracted at a 5-km grid resolution across continental France (Cf. Supplementary Table 1). To reduce collinearity and overfitting, covariates were first grouped into seven categories: bioclimatic, land cover/soil, human pressure, and host-related (deer, birds, rodents, species richness). Then, within each category, hierarchical clustering on principal components (HCPC) was applied, with clusters defined by the largest relative loss of inertia. The most relevant covariates within each cluster were selected based on literature and prior hypotheses (Cf. Table 2).

For each tick, selected covariate values were extracted within a 5 km radius around the GPS coordinates to account for environmental heterogeneity and geolocation imprecision, and the weighted median, accounting for the proportion of each cell covered by the buffer, was retained (Cf. Table 2). All covariates were centred and scaled before analysis. To further limit collinearity between selected covariates, variance inflation factors (VIFs) were assessed, using a cut-off value of 5, to remove collinear covariates (*car* package, version 3.1.3;^52^.

**Table 2:** Covariates selected for the models with their descriptions and associated hypotheses on their effects on Lyme borreliosis occurrence.

Type	Variable	Description	Resolution	Hypotheses	Source
Vectors	Habitat suitability index of *Ixodes ricinus* in France	Habitat suitability index for *I. ricinus* throughout continental France multi-criteria decision analysis integrating climate, land cover, altitude and the density of wild ungulates.	100 m	Area suitable for *I. ricinus* may be area with high density of *I. ricinus* and as such Bbsl prevalence as *I. ricinus* is the main vector species for *B. burgdorferi* sensu lato (Bbsl) in France^3,16^	^21,53^
	*I. ricinus* suitability	Proportion of suitable habitat for *I. ricinus*	1 km	Area suitable for *I*. *ricinus* may be area with high density of *I. ricinus* and as such Bbsl prevalence *I. ricinus* is the main vector species for Bbsl in France^3,16^	William Wint Mood project (undisclosed data)
Hosts	Deer	Sum of ensemble models describing the proportion of suitable habitat for roe deer, red deer, and fallow deer respectively within recorded distributions for Europe as identified from diverse source	1 km	High cervid densities are hypothesised to increase overall tick populations without directly increasing Bbsl prevalence, as cervids are incompetent reservoirs. However, their role in supporting adult tick feeding indirectly sustains nymphal tick density, which contributes to Bbsl transmission dynamics in areas with abundant reservoir-competent hosts^54^	William Wint Mood project (undisclosed data)
	Mammal species richness	Mammal species richness including major taxonomic Orders (Cetartiodactyla, Carnivora, Primates, Eulipotyphla, Chiroptera, Rodentia) and marsupials	10 km	High mammal species richness is hypothesised to elevate Bbsl prevalence in ticks by providing multiple competent hosts, which support ongoing infection cycles within tick populations^55^. However, in highly diverse communities, the “dilution effect” might reduce overall prevalence by increasing the proportion of non-competent hosts^56^.	Biodiversity mapping^57^
	Sheep and cattle density	Density of cattle and sheep per km² with weight estimated by Random Forest model with a dasymetric method	~ 10 km	Livestock grazing may affect negatively the abundance of small rodents and ticks^58^. High livestock densities are correlated with areas of human activity, potentially leading to increased reports of biting ticks	Gridded livestock of the world database^59^
	Index of rodent species richness	Predicted average number of rodent species in the field. Layer containing 5 rodent species: *Apodemus agrarius*,* Apodemus flavicollis*,* Myodes glareolus*,* Microtus arvalis*,* Apodemus sylvaticus*	1 km	Higher rodent species richness is hypothesised to elevate Bbsl. prevalence in ticks by increasing the diversity of reservoir hosts for genospecies such as *B. afzelii*. This enhances local transmission dynamics and may sustain infection cycles across various habitats^36^.	^60^
	*Myodes glareolus* suitability map	Ensemble model describing the proportion of suitable habitat for *M. glareolus*	1 km	*M. glareolus* is a good reservoir for Bbsl^61^	William Wint Mood project (undisclosed data)
	Urban tolerant bird abundance	Layer containing the sum of the predicted full year abundance of 4 species which habitat extend to anthropised areas: *Sylvia atricapilla*,* Parus major*,* Luscinia megarhynchos*,* Ficedula albicollis*	3 km	Birds that can thrive in anthropised habitat can be more susceptible to carry *B. garinii*^6^.	E-birds
	Turdidae abundance	Sum of abundance of birds belonging to the Turdidae family. Layer containing the sum of the predicted mean full year abundance of birds belonging to the Turdidae family: *Turdus iliacus*,* Turdus merula*,* Turdus philomelos*,* Turdus pilaris*	3 km	Members of the Turdidae family are reported to be hosts of *B. garinii*^62^ *Turdidae* bird abundance is hypothesised to increase Bbsl prevalence in ticks by serving as key reservoir hosts, particularly for bird-specific *Borrelia* genospecies as *B. garinii*. Their behaviour and high tick burden facilitate *Borrelia* transmission, increasing prevalence in tick populations in areas where these birds are common.	E-birds
	Passeriformes species richness	Passeriformes species richness	10 km	High passeriform species richness may be associated with more *B. garinii* prevalence^63^	Biodiversity mapping^57^
Bioclimatic	Mean temperatures of the driest quarter	Average mean temperature for the years 1970–2000 of the driest quarter of the year. A quarter is a period of three months	1 km	An association between temperature and *B. burgdorferi* sensu lato prevalence has been pointed out^7^. Mean daily temperatures during the driest quarter might modify the prevalence of *Borrelia burgdorferi* in ticks by impacting tick survival^64^, questing activity^15^, and pathogen transmission^65^. Optimal temperatures would lead to larger tick populations and longer active periods, leading to elevated *Borrelia* transmission.	Worldcilm 2^66^
	Temperature seasonality	Standard deviation of the monthly mean temperatures for the years 1970–2000	1 km	Temperature seasonality, especially shifts from colder to warmer periods, is hypothesised to increase Bbsl prevalence in ticks^7^, potentially by synchronizing host-seeking behaviour with reservoir host availability^67^, extending active seasons, and enabling differential pathogen genotype survival across varying climate^68^.	Worldcilm 2^66^
	Annual precipitation amount	Accumulated precipitation amounts over 1 year for the years 1970–2000	1 km	Higher annual precipitation is hypothesised to increase *Borrelia burgdorferi sensu lato* prevalence in ticks by supporting optimal moisture conditions and vegetation that sustain tick populations and questing behaviour^69,70^, enhancing pathogen transmission across tick-host networks.	Worldcilm 2^66^
	Mean precipitation amount of the driest quarter	Average mean precipitation for the years 1970–2000 of the driest quarter of the year. A quarter is a period of three months	1 km	Humidity plays a vital role in tick activity and survival^71^. Higher mean monthly precipitation in the driest quarter is hypothesised to increase *Borrelia burgdorferi sensu lato* prevalence by enhancing tick survival and activity during dry periods, thereby supporting sustained transmission cycles of *Borrelia* in regions prone to seasonal drought as in USA^72,73^.	Worldcilm 2^66^
Landcover	Shrubs cover fraction	Fractional cover (%) in a 100 m pixel for the shrubland class	100 m	Shrub cover fraction is hypothesised to positively influence *Borrelia burgdorferi sensu lato*, particularly *Borrelia garinii* as shrubs can provides varied resources to birds species in the form of food, nesting and shelter from predators^74^. Additionally, shrub habitats can be associated with more species richness and in some cases abundance^75,76^.	Copernicus global land service 2019^77^
	Deciduous broad leaf forest	(Discrete) forest type of layer for all pixels where the tree cover fraction exceeds 1% and matches the deciduous broad leaf forest	100 m	Deciduous forests might act as critical habitats for maintaining and amplifying *Borrelia* transmission cycles due to favourable microclimatic conditions, high host diversity, and abundant tick populations^78,79^. However, these forests’ exact role in amplifying or diluting *Borrelia* prevalence depends on the interplay between biodiversity, host availability, and habitat structure.	Copernicus global land service 2019^77^
	Grass cover fraction	Fractional cover (%) in a 100 m pixel for the herbaceous vegetation class	100 m	Grass cover fraction is hypothesised to influence Bbsl prevalence in ticks by supporting tick habitats in ecotone areas and near woodland edges, though prevalence is generally lower than in forested regions. Grasslands with adjacent habitats for reservoir hosts may contribute to localised *Borrelia* transmission dynamics.	Copernicus global land service 2019^77^
	Crops cover fraction	Fractional cover (%) in a 100 m pixel for the cropland class	100 m	Crop cover fraction is hypothesised to influence Bbsl prevalence in ticks due to the lack of suitable habitats, reservoir host diversity, and management practices in agricultural landscapes. Edge habitats near croplands, however, may support occasional tick populations and pathogen transmission, though at reduced prevalence levels compared to forested areas^80^.	Copernicus global land service 2019^77^
	Built-up cover fraction	Fractional cover (%) in a 100 m pixel for the built-up class	100 m	Built-up areas can alter the natural habitat favourable *for I. ricinus* and Bbsl reservoir, though some connected greenspaces in urban environment may support enough wildlife such as rodents and birds to support Bbsl transmission cycle^81–83^.	Copernicus global land service 2019^77^
Human pressure	Travel times cities	Accessibility to high-density urban centres in 2015 as measured by pixel-level travel times for the optimal path between each pixel and its nearest city (that is, with the shortest journey time)	1 km	Travel times to cities impact Bbsl prevalence in ticks, with higher prevalence expected in green spaces of rural areas due to greater connectivity and wildlife reservoirs. Ticks in peri-urban areas may maintain *Borrelia* transmission cycles, while urban areas further from rural habitats would exhibit on average lower prevalence due to reduced wildlife host and tick densities^84^.	^85^
	Human population density	Gridded human population estimates	1 km	Areas with high human population density may be areas with a higher probability of tick bite reports and thus tick samples than others.	Worldpop
	Human footprint	Annual dynamics of the global human footprint using eight variables that reflect various aspects of human pressures.	1 km	Human footprint is an important predictor of vector borne disease occurrence^86^. Higher human footprint index values are hypothesised to lower overall tick densities but can create isolated, fragmented habitats that maintain *Borrelia* prevalence within tick populations. Urban green spaces and edge habitats support localised *Borrelia* transmission due to the continued presence of tick hosts, even in human-modified landscapes.	^87^
	Soil biodiversity threats	The potential rather than the actual level of threat to soil organism using threats proxy (loss of above ground biodiversity, pollution and nutrient overloading, agricultural use, overgrazing, fire risk, soil erosion, land degradation, climate change)	~ 10 km	Biotic interactions within soil regulate the structure and functioning of aboveground communities and contribute to the delivery of vital ecosystem services^88,83^. As soil-dwelling arthropods, ticks can benefit from soil biota and plants for their survival^90^. We hypothesize that areas with high potential of threats to soil organism were less favourable to ticks and their hosts such as of rodents or soil dwelling birds that respectively benefit of the products of a rich soil, including seeds, fruits, and soil invertebrates.	ESDAC^91^

### Modelling

We used Generalised Additive Models (GAMs) to investigate the variation in Bbsl and Bbsl genospecies distribution, used as the response variable, in relation to the selected set of continuous explanatory covariates (see Table 1), while accounting for the spatial distribution of the observations. The general structure of the models was:

$${y}_{i}\mathrm{~Binom}\left({p}_{i}\right)\mathrm{\:where\:}logit\left({p}_{i}\right)={\beta}_{0}+{f}_{n}\left({x}_{n,i}\right)+te\left(\mathrm{l}{ong}_{i},{lat}_{i}\right)$$

where ${f}_{n}$ are spline functions applied to the explanatory covariates ${x}_{n,i}$, and $te$ is a bivariate tensor product function accounting for the spatial structure based on the longitude and latitude.

Given the large number of 23 explanatory covariates, we applied a double penalty approach to control smoothness of the model terms (i.e., curves ${f}_{n}$ and spatial surfaces $te\left(\mathrm{long},\mathrm{lat}\right)$, and perform covariate selection^92^. The first penalty controlled the complexity of smooth functions to prevent overfitting by ensuring that relationships remained smooth and interpretable. The second penalty shrunk entire uninformative smooth functions towards zero and effectively removing them from the model. The degree of smoothing was selected using the restricted maximum likelihood (REML) method, and the number of basis functions for smooth terms was limited to the default of 10 simple terms to avoid overfitting.

Three GAM specifications were fitted. The first model (M0) investigated factors associated with the presence of Bbsl across all observations, encompassing both presence (${y}_{i}=1$) and absence (${y}_{i}=0$) of Bbsl genospecies. In a second step, we focused the modelling process to locations where a *Borrelia* genospecies was detected, to identify factors associated with the infection probability of specific genospecies. Only *B. afzelii* and *B. garinii* had sufficient data for the whole territory, as they were the most frequent genospecies. Therefore, two separate GAMs were constructed (M1 for *B. afzelii* and M2 for *B. garinii*), using the subset of 285 individuals where a Bbsl genospecies was present. In these models, infection with *B. garinii* or *B. afzelii* (depending on the model) was coded as presence (${y}_{i}=1$), while infection with another Bbsl genospecies was coded as absence (${y}_{i}=0$).

The predicted prevalence of *B. afzelii* and *B. garinii* was calculated by the product of the predicted general Bbsl presence (M0) with the species-specific relative probability (M1 or M2 depending on the genospecies).

Model fitting was performed using the *mgcv* package (v1.9.1;^93^). All final models were checked for residuals validity using the Dharma package. (v0.4.7 ;^94^).

In addition to GAMs, we used spatial relative risk analysis to identify areas of elevated risk (i.e., “hot spots”) where tick infection by Bbsl and its genospecies was higher than expected, while accounting for underlying tick distribution. Spatial relative risk was estimated using the *sparr* package in R (v2.3.15;^95^). This method estimates the relative density of pathogen presence versus absence points. To account for our spatial sampling, we applied an adaptive bandwidth, allowing finer resolution in densely sampled regions and smoother estimates in sparsely sampled areas. The relative risk surface was estimated asymmetrically, meaning that different bandwidths were applied to the case and control point distributions, as recommended by Davies and Hazelton^96^. Edge effects were corrected using the default settings described by Diggle^97^. Statistical significance of elevated and diminished risk areas was assessed using asymptotic tolerance contours based on p-values, generated via the Monte Carlo method with 1,000 simulations.

All analyses were performed in R version 4.3.2^98^.

## Supplementary Information

Below is the link to the electronic supplementary material.

Supplementary Material 1

## Data Availability

The dataset analysed during the current study is available in the Data Gouv repository, https://doi.org/10.57745/FIRZ0C.
